# The Virulence and Specificity of Tuberculosis

**Published:** 1868-12-01

**Authors:** J. A. Villemin

**Affiliations:** Professor of the School of Val de Grâce


CHICAGO MEDICAL JOURNAL.

*Vol. XXV.--DECEMBER* 1, 1868.—*No.* 23.

### THE VIRULENCE AND SPECIFICITY OF TUBERCULOSIS.

*Read before the Academy of Sciences* (*Paris*) *by* DR. J. A. VILLEMIN, *Professor of the School of Val de Grâce.*

TRANSLATED EXPRESSLY FOR THIS JOURNAL, BY WALTER HAY, M.D., ASSOCIATE EDITOR.

(*Continued from page* 729.)

THUS, then, far from denying any significance to experimental facts long since established, and refusing to perceive analogies the most striking, it must be conceded to us that our inoculations of tubercle have been made under conditions entirely similar to those which have accompanied most experiments of similar character; and, even, in comparison to those of glanders, the corresponding circumstances are multiplied to the degree of constituting an association of phenomena almost identical. Here are undeniable facts—such that no contradiction, however urgent it may be, can refute. It is next asked, where is the serum, regarded as necessary to the constitution of a virus, which should be in the granulations of glanders, the tumor of farcy, the froth of the solipeds, and which is not found in tubercle, nor in the expectoration of phthisical subjects. Moreover, what is known of the physical condition of contagium? Is it solid, liquid, or gaseous? Do not the brilliant and judicious experiments of M. Chauveau demonstrate to us, on the contrary, that certain virulent fluids have no activity unless they contain solid corpuscles?

In spite of all the efforts attempted to deny to tubercle the properties of virulent substances, no one has, however, been able to refuse recognition to the remarkable power, which its inoculation exercises, of involving the formation, throughout the economy of tubercular productions in great number, and disseminated through the most distant organs. Now, what is comparable to this phenomenon, unless it be the inoculation of virus? However, an analogy as evident has been disputed, and we are reproached with having created at once two difficulties: "the first of which is to establish the existence of the virulent principle; the second to explain how it engenders tuberculous matter." We believe that there are no other proofs of the virulence of a pathological product than its inoculability; and, as to explaining how tuberculous virus engenders tubercle, the difficulty is neither more nor less great than to say how the virus of glanders engenders the tubercle of glanders, or syphilitic virus, syphilitic gummata. We have already stated elsewhere, that the relations between any phenomenon whatsoever and its cause establish, but do not explain themselves.

It is by virtue of a *catalytic* action, analogous to that of ferments, say some, that inoculated tubercle infects the economy with tubercle. Well, has not virus been compared to ferment, and does not the name *Zymotic*, given to virulent maladies, confirm this analogy?

Next, it is asserted that inoculated tuberculous matter acts neither in the manner of a virus, nor by catalysis; it is by a process analogous to that of fecundation. Doubtless it might be asked if, at the termination of the process of fecundation, the fecundating principle reproduces and multiplies itself—if sperm or pollen are recovered from the subject fecundated; but it does not imply the identity of all the terms. Morever, if there is in this case a sort of fecundation, it exists likewise in the case of all other virulent substances.

By inoculation the detritus of a chancre gives chancre, the variolous pustle studs the skin with variolous pustules, the tubercle of glanders disseminates glander tubercles throughout the organs, just as the phthisical tubercle infects the system with phthisical tubercles.

This hypothesis of fecundation is but one mode of apprehending and explaining the action of virus. It is no less ingenious than all the rest which have been proposed hitherto, and, as we shall hereafter see, tuberculosis is not the only disease to which it has been applied. But in the present state of science, I do not perceive any advantage in substituting for the word inoculation, that of fecundation, applied to this fact of the transmission of a disease from one individual to another by means of a particle of morbid matter. If, instead of seeking our comparisons in unexplained chemical processes and in very remote physiological acts, we form them in the ranks of similar facts — if we observe, for example, what occurs in inoculations of glanders, a disease so similar to tuberculosis, what do we see? We determine that all the particulars observed in the inoculation of tubercles are recognizable in the inoculations of glanders.

These are, by their physical characters, the same inoculated matters, the same alterations of the ganglia and of the lymphatic vessels, the same anatomical processes generalized in the viscera, with preference for the respiratory organs. If, when there is introduced into a wound some of the liquid of the froth, or a little of the caseous matter of a tubercle of glanders, or the detritus of a farcy-button, there is produced at the end of a few days, at the point of inoculation, a little tumor which frequently ulcerates; then from this tumefaction there arises a cord, which extends to the hypertrophied hard and painful ganglia.* Usually, if the inoculation has been performed on one side only, the corresponding ganglia are alone diseased.

*Bouley, *Bulletin de l'Academie de Medicine*, 1838–39, p. 793.

These ganglia incised appear full of glander tubercle, and the lymphatic vessels reach their parietes infiltrated with the same product. There is a series of lesions which is reproduced, in a manner exactly parallel, in inoculations of phthisis. Coincidently with these local alterations there are established in the lungs, the respiratory mucous membrane, the liver, the testicles, the intestines, etc., nodules of variable size and number.* Are there in pathology two processes which have between themselves more of striking analogies than these results of the inoculation of glanders and of tubercle?

In syphilis, is there not also something comparable? The ganglionic plexus manifests to us the part performed by the lymphatic system in immediate proximity to the virulent matter.

Thus the objections which have been urged, in regard to the inoculated substance, to the local accidents of inoculation, to the mode of generalization, to the anatomical lesion, etc., apply as well to glanders as to tuberculosis; and, curious coincidence, these same objections were, in fact, made at the time when the inoculability, the virulence and the specificity of glanders was discussed, just as now. When we established in a work, published recently, a parallel between tuberculosis and farcy-glanders, and when we exhibited these numerous analogies which exist between these two affections, we certainly did not doubt that these analogies would be completed and continued by the similarity of the arguments invoked against the virulence of both of these diseases.†

But before proceeding farther, we must be permitted to correct an error which has insinuated itself into some minds. We are credited with the opinion that glanders and tubercle are identical. We have never thought so. The chapter of our book, in which we have established the approximations of these two affections, has for its title: *Glanders is the disease approximating nearest to Tuberculosis.* We have here devel-

* Saint-Cyr., *loc. cit.*, p.65.

† Villemin, *Etudes sur la tuberculosis.* Paris, 1868, p. 431.

oped the affinities which exist between these two morbid entities, as might be done, I think, between scarlatina and rubeola, whilst maintaining at the same time the complete and essential separation between the two elements of the comparison. It must not be imagined that the inoculation and the virulence of farcy-glanders was admitted without opposition. The experiment created as great an excitement as did that of tuberculosis. It was vigorously contested in the consequences and even denied radically. Veterinary physicians were for a long time divided into two opposing factions: the contagionists and the non-contagionists. When Gohier, in 1813, had announced the results of his experiments of inoculation, the non-contagionists showed themselves fertile in expedients. At first they denied the fact, and opposed their own negative results to the positive results of their adversaries. They even denied the possibiltty of the fact. "We ask next, said Dupuy, how *solid matter*, such as that which constitutes the tubercle (of glanders) could become contagious."* It has been more than fifty years since this argument was adduced against glanders, and now we behold it resuscitated against phthisis. It was especially against the chronic form of glanders which approximates most closely to phthisis, that attacks were directed. And what reasons were made available against its contagiousness and its virulence? The same, *identically the same*, as those which have been adduced against the virulence of tuberculosis. Hear Delafond:† "Among the numerous diseases, of chronic type, of our domestic animals, do we discover a single one which is evidently contagious? We know not one. Now, why should glanders be made an exception? We ask, is it possible to find, amongst all the characteristics of this disease, a single one which may be compared with those, so very numerous, which mark diseases positively contagious? No.

All contagious diseases are of acute, or subacute type; the

* Dupuy, *loc. cit.*, p. 455.

† Delafond. *Traite sur la police sanitaire des animaux domestiques.* Paris, 1839, p. 603.

symptoms which designate them are constant, unequivocal, their progress is rapid, their duration brief; their termination, although often variable, is generally unfavorable; all have a known palpable *virus*, transmitting the disease by inoculation. Now it is exactly the opposite characteristics which appertain to glanders."

By the partizans of these theories, glanders, like the phthisis of our adversaries, is attributable to no other causes than diminished action, resulting "from long sustained and very exhausting fatigue....from alimentation maintained for a long time with a diet invariable or innutritious....from residence in cold, damp, badly ventilated and badly lighted localities...."from arrest of perspiration"....from long continued suffering from the presence of chronic diseases, whether internal or external, from resorptions of morbid matters of whatsoever kind, which occur during the course of many diseases.*

Contagion from the horse to man seems not to be sufficiently convincing. Who does not remember the brilliant contest of 1836? And two years later, Delafond refuting Rayer, exclaims again: "No, the cause of the disease called farcy *is not specific.* We recognize in it only the result of an infection, originating in animal matter, fixed or volatile, altered by the presence of air, which, introduced into the economy by absorption, determine morbid effects so much the more intense as the subjects are more debilitated and already predisposed to putrid infection.*

But when crushed by the evidence, the noncontagionists could no longer resist the power of facts, they change their batteries. "Of what significance," said they, "is the inoculation of the product of glanders, and what becomes of its virulence and specificity, since other substances communicate glanders as well as this — since this disease can even be provoked by simple wounds?"

Dupuy, separating chronic from acute glanders, which he

*Delaford, *loc. cit.*, p. 595.

*Id., *loc. cit.*, p. 684.

calls *gangrenous coryza*, induces this latter upon healthy horses by inserting under their skins a *fragment of spleen* taken from a horse dead in consequence of section of the pneumogastric nerve ;* animal substances undergoing putrefaction, such as blood, portions of muscles, etc., will produce the same effect, according to him.†

He induced glanders also in horses by inoculating them with the puriform matter which flowed from the nostrils of a horse not affected with glanders.‡

Renault subjected to the examination of the Academy of Medicine pathological preparations attesting the production of glanders by injections of pus, not even suspected,‖ and in order to demonstrate the reality of this glanders, he inoculated it successfully into healthy horses.§ He published detailed observations of farcy-glanders, originating as a sequel to a simple *sore throat*, from a *contusion* of the upper eyelid, from a *fistule* of the spermatic cord, consecutive to castration.*a

Finally, Dupuy demonstrated that *setons* passed through the shoulders of horses had induced in them glanders.†b

In Germany, Erdt developed glanders in four horses by inoculating them with the pathological products of scrofula (1834). Are not all these experiments, all these assertions, all these arguments, directed against the virulence and the specificity of glanders with as much authority and force as those which are opposed to tuberculosis. If then it is desired to maintain them in order to prevent the admission of tuberculosis into the list of virulent maladies, farcy-glanders must also necessarily be eradicated therefrom, being retained therein with no more propriety than the latter.

* Dupuy. Bull. de l'Academie de Medicine, 1836, p. 481.

† Dupuy, *de l'affections tuberculeuse.* Paris, 1817, p. 244.

‡ Ibid, p. 454.

‖ Renault. *Bulletin de l'Académie de Médicine*, 1839, p. 69. 1840, p. 402.

§ Renault. *Recueil de Médicine Vétérinaire*, 1840, p. 257.

*a Renault. *Recueil* de Medicine Veterinaire, 1835. p. 393.

†b Dupuy. Bulletin de l'Academie de Medicine, 1836. p. 481.

The objections urged (I will not say to the inoculation of tubercle, for that is undeniable, but to its virulence and specificity,) have been made not only to glanders, its congener, but to syphilis, its relation one degree farther removed. Syphilitic virus has had, like that of glanders and that of tuberculosis, its violent enemies, and the arms of which they made use are no other than those which have been gathered up again by the adversaries of the specificity of tuberculosis. And, moreover, the first argument was to deny its inoculability and to oppose, to positive experimental results, those which were negative and contradictory. This is what was done by Bru, and in order to account for venereal accidents, he admitted, *un mode venerien*, explained physically by electricity.* Caron, following the footsteps of Bru, assimilated the transmission of syphilitic accidents to the *impregnation* of females; he explaned this by the *fecundation* and not by the absorption and the multiplication of a virus. "It is not," said he, "a virus which is inoculated in the venereal contagion, but it is an occult vice which is developed in us, it is nature, it is life, which establishes the syphilitic constitution." The venereal infection has at first only a local action, which *extends itself successively to certain parts.*† This theory of fecundation is already old, as we perceive, and with it we recognize at the same time the mode of progression, step by step, by which it is attempted to explain the development of tubercles in the organs. Jourdain likewise denied the existence of syphilitic virus, by denying the specificity of the venereal disease, and all significance to its inoculation. He affirmed that "similar and even more grave results are seen to result from a simple puncture.‡ The constitutional manifestations of syphilis depended, according to Jourdain, upon a great number of different causes. Richon-des-Brus,

*Bru. *Nouvelle methode de traiter les maladies veneriennes par les gateaux mercuriels.* Paris, 1789.

†Caron. *Nouvelle doctrine des maladies vneriennes.* Paris, 1811.

‡Jourdain. *Waite complet des maladies veneriennes.* Paris, 1816.

in his book, "The non existence of the venereal virus,"* and Desruelles continued the war against the virulence and specificity of syphilis.

The first admitted the spontaneity of this malady, and the second sought its causes in the seasons, the temperature, hygrometry, etc.

Thus, then, there was a time when glanders and syphilis, like the tuberculosis of our opponents, originated from every thing — was inoculated by every thing.

What is to be learned from these teachings of history? It is that the facts which have suggested such analogous arguments and contradictions must necessarily have among themselves very great analogies of character. In fact, syphilis, glanders and tuberculosis form a nosological group whose species have among themselves incontestable affinities.

If tuberculosis is specific and virulent, it is for that very reason contagious. But it remains to determine the mode and the conditions of its transmissibility. That phthisis may be communicable by inoculation like syphilis, I believe to have been placed experimentally beyond any doubt. It is inoculable from man to certain animals, and from these animals to others of the same species. Is it from man to man? It is absolutely forbidden to apply the experimental test. But every thing indicates the affirmative. To say that it is not so because no one has seen an instance of it, is to refuse to the inoculability of tubercle the right to appear in its day and hour like every other scientific truth. A discovery consists exactly in the summing up of the evidence of a fact which had up to that time eluded observation. At the first report of human glanders, for example, it might have been objected that the thing had never been seen, who then had observed the relations of the phlegmasiæ of cardiac serous membranes with articular rheumatism before the beautiful discovery of the *law of coincidences.*

On the other hand the transmissibility of tuberculosis by the act of cohabitation, although regarded as very probable by

* Richon-des-Brus. *De la non existence du virus venerien.* Paris, 1826.

a certain number of distinguished practitioners, is not, however, so evident that it may not be contested. There is in this matter an obscurity which imposes the greatest reserve, and consequently the clinical evidence, especially in a large hospital, is not sufficient to justify the absolute denial of this mode of transmission of phthisis. Large cities, vast establishments where so many maladies are accumulated, and which are impressed with so many morbific matters, are far from being favorable for the especially difficult study of contagion. In these localities, for instance, the transmission of typhoid fever is almost always concealed, whilst it becomes apparent in small villages, in the bosom of families nearly isolated, to the observer placed in circumstances less complex, more clear, more exact, and on that account more conclusive. And then, moreover, what is the communicable disease whose transmissibility has not been determined ? May we not then believe the clinical observer authorized to affirm every where and always the spontaneity of tuberculosis and to deny absolutely the possibility of its transmission by contamination ? But would it be right, on this account, that he should infer nothing against the inoculability of tucerculosis ? It would, therefore, be much more in accordance with modesty that the solution of questions relative to the propagation of tuberculosis in the human species should be reserved for the future, which will decide, we may be sure, not upon argument and theories, but upon positive and perfectly demonstrated facts. For myself, aided by experiment, I have sought to determine the circumstances which appeared to me to play a preponderating part in the transmission of phthisis.

The curious and important results which I have already obtained, seem to me to be destined to throw some light upon this point. If the Academy will grant me the permission, I shall have the honor to communicate them to it hereafter.

I conclude, gentlemen, by thanking the Academy for the courtesy with which it has condescended to hear the explanations which I have just submitted to it; I am aware, however, that I owe it entirely to the importance of the subject which I have treated.

